# A Grade I Intracranial Meningioma with Metastasis to Multiple Vertebral Bodies: A Case Report and Literature Review

**DOI:** 10.7759/cureus.11477

**Published:** 2020-11-13

**Authors:** Renee Hanna, Aharon M Feldman, Christian E Keller, M. Salim Siddiqui

**Affiliations:** 1 Radiation Oncology, Henry Ford Health System, Detroit, USA; 2 Pathology, Henry Ford Health System, Detroit, USA

**Keywords:** meningioma, who grade i, brain tumor, metastasis

## Abstract

World Health Organization (WHO) grade I meningiomas are slow-growing and typically benign brain tumors that can often be easily removed by surgery and rarely become malignant. We report the case of a WHO grade I meningioma in a 67-year-old man with multiple extracranial metastases.

## Introduction

Meningioma is a benign tumor arising from the arachnoid cells of the leptomeninges and is one of the most commonly diagnosed primary brain tumors in adults [1]. Distant metastasis is rare and is generally seen only in World Health Organization (WHO) grade II and III tumors. Meningiomas are usually classified based on their dural site of origin as well as the involvement of adjacent tissues. Many remain asymptomatic, but meningiomas may present clinically with focal or generalized seizure disorders or neuropsychological decline [2].

## Case presentation

**Figure 1:**
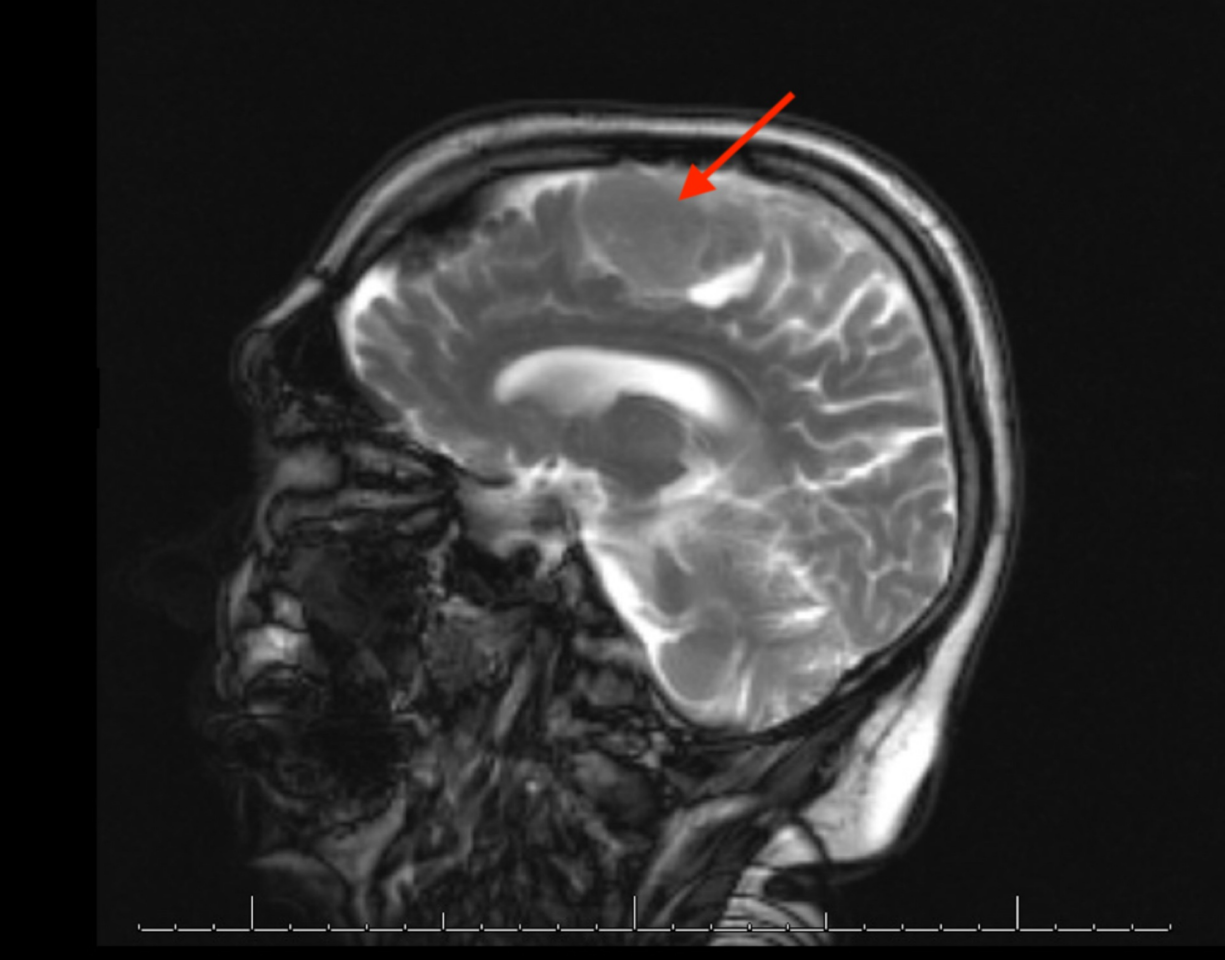
Mass measuring 5.5 x 3 x 3 cm, abutting and likely invading the midline superior sagittal sinus and the premotor and motor strip on the left. Heterogeneous enhancement with areas of somewhat decreased signal noted within it. Lobulated, with fairly well-defined margins.

A 67-year-old white man was diagnosed with an incidental parasagittal meningioma in the left frontoparietal lobe measuring 4 x 2 x 2.5 cm on imaging. He initially declined surgical resection and opted for observation. On reimaging six years later, the tumor had grown to 5.5 x 3 x 3 cm and had extended to the superior sagittal sinus and the left premotor and motor areas (Figure 1). Eight years after initial diagnosis, the patient noted a decline in cursive handwriting and subsequently was treated with external beam radiation therapy to a dose of 54 Gy in 30 fractions. Afterwards the patient noted return of his baseline handwriting.

Two years after completion of the external beam radiation therapy, the patient presented with a sudden transient episode of aphasia. An MRI of the brain showed a slight interval increase in the size of the mass, and he underwent a left frontoparietal craniotomy. Pathologic examination revealed a WHO grade I meningioma.

**Figure 2:**
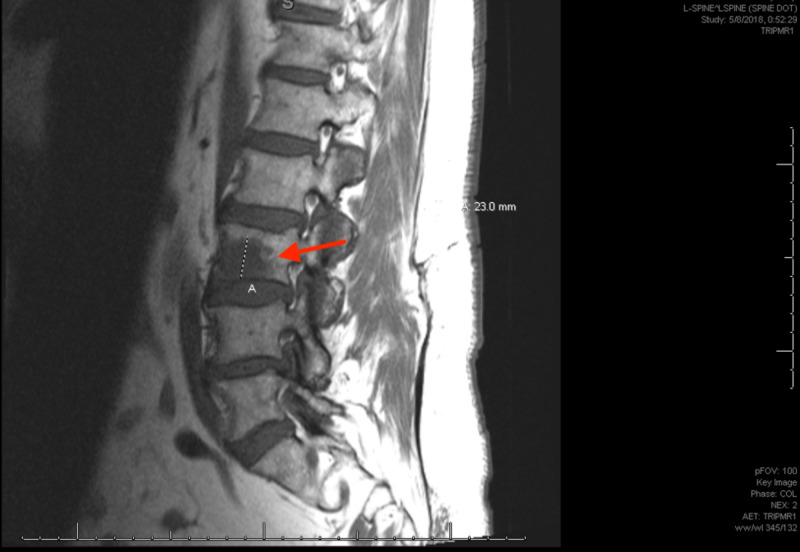
MRI of the spine revealing lytic lesions at L3.

**Figure 3:**
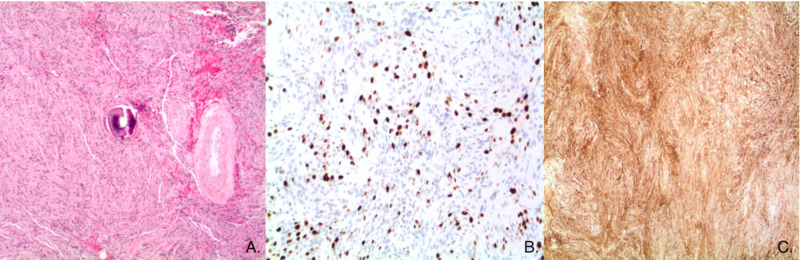
A: (Hematoxylin and eosin stained section, original magnification 40x): The neoplastic cells are arranged and short, randomly oriented fascicles and focally whorls. In the center of the image, a psammoma body is seen next to a hyalinized blood vessel. The neoplastic cells have ill-defined cells borders (so-called ‘syncytial growth pattern’) and abundant eosinophilic cytoplasm. The nuclei of the neoplastic cells are relatively uniform and predominantly fusiform. There is no loss of architecture (so-called ‘sheeting’), small cell formation, nuclear pleomorphism or tumor necrosis noted. Invasion of neocortex cannot be evidenced in the sample. There is no increase in mitotic activity. B: (MIB-1, Ki67 immunohistochemical stain, original magnification 100x): The cell cycle marker Ki67 is expressed in approximately 10% of the neoplastic cells, which corroborates that low mitotic index. C: (Epithelial membrane antigen [EMA] immunohistochemical stain, original magnification 40x): The neoplastic cells strongly and diffusely express EMA. This finding supports meningothelial differentiation.

The patient experienced recurrence with increase in size of the meningioma with new bilateral temporal lobe lesions, and was subsequently treated with fractionated stereotactic radiosurgery (SRS). Shortly after completing SRS, he presented with persistent lower back pain. An MRI of the spine with and without contrast revealed lytic lesions in the C4, T11, and L3 vertebrae, suspicious for malignancy (Figure 2). A biopsy of the L3 lesion revealed clusters of proliferating meningothelial cells admixed with hematopoietic cells and visible mitotic figures with immunomorphological features consistent with a WHO grade II meningioma (Figure 3).

**Figure 4:**
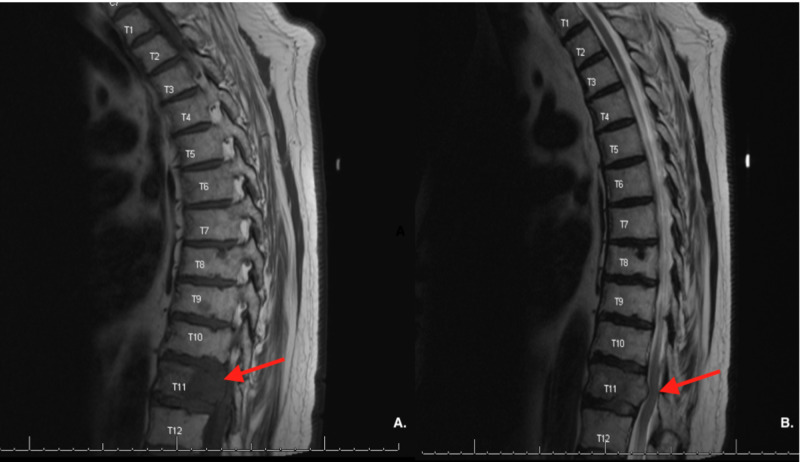
A: MRI of the lumbar spine revealing lytic lesions at T11. B: moderate compression of thoracic canal and spinal cord by mass.

The patient was subsequently treated with SRS to the right temporal tip lesion adjacent to the dura and the T11 lesion. Subsequently, he complained of left-sided mid-thoracic pain radiating down his thigh, difficulty with ambulation, and stool incontinence, with MRI consistent with spinal cord compression (Figure 4). He was again treated with SRS to the C2, C4, and L3 lesions. One month later, the patient was admitted to the emergency department for severe sepsis and was noted to have a new 7 cm mass on the superior aspect of scalp, suspicious for metastasis. The patient died two days later.

## Discussion

Surgical resection with the goal of a gross total resection is the mainstay of treatment of meningiomas [3,4]. Radiation therapy is an acceptable alternative for patients who are not surgical candidates or who decline surgery [3]. Though extracranial metastasis is exceedingly rare for meningiomas, with an occurrence of fewer than 1 per 1000 cases [5], recent analyses have determined that the prevalence of metastatic meningioma may be underreported in the literature [6]. The rarity of grade I metastatic meningioma may also simply be due to diagnostic error, reflecting incompletely sampled higher-grade meningiomas mistakenly diagnosed as grade I.

Pathogenesis of grade I meningioma metastasis includes tumor invasion of the venous sinuses [7,8] and surgical seeding [9]. The most common sites of extracranial metastases for metastatic meningiomas are lung, liver, lymph nodes, and bone [10]. We identified five cases of metastatic WHO grade I meningiomas in the literature that describe metastases to the lungs [11-13], multiple vertebrae, the retroperitoneum, cervical lymph nodes, and the right iliac wing (Table 1) [12-15]. Therapy for these rare cases has included a combination of gross total resection, radiotherapy, and chemotherapy. There are currently no US Food and Drug Administration approved chemotherapy regimens for meningioma; but in 2011, the National Comprehensive Care Network released guidelines advocating for the use of interferon alpha, somatostatin receptor agonists, and vascular endothelial growth factor signaling inhibitors for refractory meningioma cases [16,17]. Immunohistochemical findings point to other possible therapeutic avenues such as targeting insulin-like growth factor-1 (IGF-1) receptor, epidermal growth factor receptor (EGFR), and growth hormone receptor (GHr). Presence of these receptors was conserved across all histological grades and was found in 88% to 94% of meningiomas in a recent study by Baxter et al. [18].

**Table 1:** Reported cases of diagnosed World Health Organization (WHO) grade I meningiomas with associated extracranial metastasis IMRT: intensity-modulated radiotherapy; EBRT: external beam radiation therapy.

Reference	Article Type	Age (years)/ Sex	Initial Presentation	Initial Location	WHO grade	Metastasis	Treatment	Outcome
Woo et al., 2019 [11]	Case Report	37/F	2-month history of progressive blurred vision and papilledema	L frontal lobe, parasagittal	I	R lower lung lobe	Gross total resection, radiotherapy (50.4 Gy)	Disease remission
Erman et al., 2005 [12]	Case Report	34/F	Not specified	L frontal lobe, parasagittal	I, II, III	Both lungs	Gross total resection, radiotherapy, chemotherapy	Patient died in Intensive Care Unit as a result of respiratory failure
Lee et al., 2009 [13]	Case Report	68/M	2-week history of L sided motor weakness and dysarthria	R lateral ventricle	I, II (recurrence)	Spine: T5, T10, L1, L3, L4, S1, S2, T7, Retroperitoneum, both lungs	Gross total resection, radiation therapy, Decompressive total laminectomy of T7 and subtotal T6 with removal of the epidural mass	Died several months later
Moubayed et al., 2011 [14]	Case Report	58/M	Not specified	L frontal lobe	I, III	Cervical lymph nodes	Lymph node excision, 2 radiation treatments, 60 Gy IMRT then 70 Gy IMRT to ipsilateral neck	Disease remission
Azene et al., 2016 [15]	Case Report	69/F	Not specified	R frontal lobe, parafalcine	I, II	R iliac wing	Two near total resection, 34 fractions EBRT	Not specified

The possibility of extracranial metastasis presents challenges when considering the appropriate observation practices for patients with grade I meningioma, with some authors advocating for full body CT imaging as an option for extracranial examination [19]. Recent studies have also found value in whole-body positron emission tomography/computed tomography (PET/CT) using either fluorodeoxyglucose (FDG) or 68Ga-DOTA-octreotate (DOTATATE) tracers. These were especially recommended for those with symptomatic lesions suggestive of metastasis or asymptomatic patients with greater than two recurrences [20].

## Conclusions

Grade I meningioma has been recognized as one of the most common intracranial neoplasms, with only a few cases of metastasis described. In patients presenting with aggressive disease or multiple recurrences, further investigation into potential immunohistochemical targets that could signal tumor malignancy should be considered. Checking molecular signatures that herald aggressive behavior may also help prevent repercussions due to diagnostic error.

## References

[1] Baeesa SS, Hussein D, Altalhy A, Bakhaidar MG, Alghamdi FA, Bangash M, Abuzenadah A (2018). Malignant transformation and spine metastasis of an intracranial grade I meningioma: in situ immunofluorescence analysis of cancer stem cells case report and literature review. World Neurosurg.

[2] Whittle IR, Smith C, Navoo P, Collie D (2004). Meningiomas. Lancet.

[3] Day SE, Halasz LM (2017). Radiation therapy for WHO grade I meningioma. Chin Clin Oncol.

[4] Rogers L, Zhang P, Vogelbaum MA, Mehta MP (2018). Erratum. Intermediate-risk meningioma: initial outcomes from NRG Oncology RTOG 0539. J Neurosurg.

[5] Adlakha A, Rao K, Adlakha H, Perry A, Crotty TB, Scheithauer BW, Ryu JH (1999). Meningioma metastatic to the lung. Mayo Clin Proc.

[6] Surov A, Gottschling S, Bolz J (2013). Distant metastases in meningioma: an underestimated problem. J Neurooncol.

[7] Enomoto T, Aoki M, Kouzakim Y (2019). WHO grade I meningioma metastasis to the lung 26 years after initial surgery: a case report and literature review. NMC Case Rep J.

[8] Hu S, Zhang Y, Sun Y (2018). Lung metastases from intracranial bleeding meningioma: a case report. Medicine.

[9] Kok DL, Hendry S, and Alvarez B (2018). Iatrogenic subcutaneous metastasis from WHO Grade I intracranial meningioma. J Clin Neurosci.

[10] Asioli S, Senetta R, Maldi E, D’Ambrosio E, Satolli MA, Bussolati G, Cassoni P (2007). "Benign" metastatic meningioma: clinico-pathological analysis of one case metastasising to the lung and overview on the concepts of either primitive or metastatic meningiomas of the lung. Virchows Archiv.

[11] Woo PYM, Hung RSL, Takemura S, Chan KY, Kwok JCK (2019). Pulmonary metastasis from a World Health Organization grade I intracranial parasagittal meningioma: a case report. Hong Kong Med J.

[12] Erman T, Hanta I, Haciyakupoglu S, Zorludemir S, Zeren H, Göçer AI (2005). Huge bilateral pulmonary and pleural metastasis from intracranial meningioma: a case report and review of the literature. J Neurooncol.

[13] Lee GC, Choi SW, Kim SH, Kwon HJ (2009). Multiple extracranial metastases of atypical meningiomas. J Korean Neurosurg Soc.

[14] Moubayed SP, Guertin L, Lambert C, Desrochers P, Nehme J, Coulombe G (2013). Successful treatment of anaplastic meningioma metastatic to cervical lymph nodes. Head Neck.

[15] Azene EM, Gai QW, Tabar SP, Morrison AL, Meisenberg B (2008). Metastasis of a histologically benign-appearing meningioma to the iliac bone. J Clin Oncol.

[16] Moazzam AA, Wagle N, and Zada G (2020). NCCN clinical guidelines in oncology. Neurosurg Focus.

[17] Kessler RA, Garzon-Muvdi T, Yang W (2017). Metastatic atypical and anaplastic meningioma: a case series and review of the literature. World Neurosurg.

[18] Baxter DS, Orrego A, Rosenfeld JV, Mathiesen T (2014). An audit of immunohistochemical marker patterns in meningioma. J Clin Neurosci.

[19] Fabi A, Nuzzo C, Vidiri A, Ciccarese M, Felici A, Cattani F, Cognetti F (2006). Bone and lung metastases from intracranial meningioma. Anticancer Res.

[20] Dalle Ore CL, Magill ST, Yen AJ (2019). Meningioma metastases: incidence and proposed screening paradigm. J Neurosurg.

